# New Rubber

**Published:** 1868-01

**Authors:** 


### Editorial.

### NEW RUBBER.

THE profession has recently been made aware, to some extent at least, of the fact, that a gum for Dental purposes, manufactured under a patent different from that of the Goodyear patent, and in no wise infringing the latter, is about being introduced to the profession.

This, we trust, will bring matters to a crisis between the Goodyear Dental Vulcanite Company and the Dentists. Said company has pursued a very unjust and tyrannical course in this matter, and the Dentists are determined to protect themselves from its outrageous demands, even if necessary, to the abandonment of rubber entirely in Dental practice. A few have been made to succumb to the demands of this company, by the fraud, threats, deception and lying practiced by their agents. The most abundant evidence of this can be adduced at any time.

The course of their agents wherever they go, is such as no just cause would require; for instance, one doing a medium business in a neighboring city, writes us, that they demand of him six hundred dollars for a license to use rubber for the present year, 1868; another with more business, says they propose to charge him two hundred dollars; to others they propose for much less. The circumstances connected with these localities, cause these variations.

Quite as great and unequal differences have been made in the demands of settlement for the past use; in many cases a mere moiety being accepted as an inducement to effect a settlement, in order that other parties might be influenced to settle. Had this company been disposed to act honorably and justly with the profession they would have realized largely, and met with no opposition, except perhaps an occasional protest.

But such is the opposition and determination now aroused in the profession, that no terms the company can make will be accepted.

We are led to this conclusion simply from our acquaintance with the feelings of the profession, especially of the West, upon this subject.

The rubber referred to above, is manufactured under a patent issued to Edwin L. Simpson, of Bridgeport, Conn., and manufactured by A. R. Hale, of that city; who guarantees to defend all persons using his gum, in all prosecutions brought against them, at his own cost.

D. H. Porter, of Bridgeport, is the general agent for the sale of this gum. His notice will be found in the advertising sheet of this Journal, to which we refer the reader.

We have hitherto refrained from discussing this subject in the pages of the REGISTER. It is one distasteful to ourselves, and we presume, so to a certain extent to our readers, and yet the time has come, when something must be said; and when we write upon this subject, we hope our readers will consider it is from necessity and not from choice. T.

